# Novel Insights Into the Mechanisms of Abdominal Pain in Obstructive Bowel Disorders

**DOI:** 10.3389/fnint.2018.00023

**Published:** 2018-06-08

**Authors:** Xuan-Zheng Shi, You-Min Lin, Shrilakshmi Hegde

**Affiliations:** Department of Internal Medicine, University of Texas Medical Branch, Galveston, TX, United States

**Keywords:** mechanical stretch, abdominal pain, gene transcription, motility, visceral sensitivity, smooth muscle, sensory neurons

## Abstract

Obstructive bowel disorders (OBD) are characterized by lumen distention due to mechanical or functional obstruction in the gut. Abdominal pain is one of the main symptoms in OBD. In this article, we aim to critically review the potential mechanisms for acute and chronic pain in bowel obstruction (BO). While clustered contractions and associated increase of intraluminal pressure may account for colicky pain in simple obstruction, ischemia may be involved in acute pain in severe conditions such as closed loop obstruction. Recent preclinical studies discovered that visceral sensitivity is increased in BO, and visceral hypersensitivity may underlie the mechanisms of chronic abdominal pain in BO. Mounting evidence suggests that lumen distension, as a circumferential mechanical stretch, alters gene expression (mechano-transcription) in the distended bowel, and mechano-transcription of nociceptive and inflammatory mediators plays a critical role in the development of visceral hypersensitivity in BO. Mechano-transcription of nerve growth factor (NGF) in gut smooth muscle cells is found to increase voltage-gated Na^+^ channel (Na_v_) activity of the primary sensory neurons by up-regulating expression of TTX-resistant Na_v_1.8, whereas mechanical stretch-induced brain-derived neurotrophic factor (BDNF) reduces K_v_ currents especially A-type (IA) currents by down-regulating expression of specific IA subtypes such as K_v_1.4. The NGF and BDNF mediated changes in gene expression and channel functions in the primary sensory neurons may constitute the main mechanisms of visceral hypersensitivity in OBD. In addition, mechanical stretch-induced COX-2 and other inflammatory mediators in the gut may also contribute to abdominal pain by activating and sensitizing nociceptors.

## Introduction

Obstructive bowel disorders (OBD) are characterized by lumen distention due to mechanical or functional obstruction. OBD lead to considerable morbidity and mortality in adults and children (Welch, 1990; Cappell and Batke, 2008; Wells et al., 2017). Mechanical bowel obstruction (BO) is the prototype of OBD, and constitutes more than 300,000 admissions annually in the United States alone. It is responsible for nearly 30,000 deaths and direct costs of more than $3 billion in medical care annually (Milenkovic et al., 2006; Cappell and Batke, 2008). Mechanical obstruction is caused by numerous factors, and may originate outside to the intestine (e.g., adhesions) or inside (e.g., carcinoma and diverticulitis). Small bowel obstruction (SBO) is generally caused by benign lesions such as adhesions, whereas large bowel obstruction (LBO) is often caused by cancers. Mechanical obstruction may be further categorized as partial (when gas or liquid stool can pass through) or complete (when no contents can pass), and simple (one-end obstruction) or closed loop (two-end obstruction). Complete, especially closed loop obstruction requires surgery, whereas partial simple obstruction is often managed conservatively (Cappell and Batke, 2008).

Functional obstruction results from neuromuscular dysfunction of the gastrointestinal (GI) tract, such as in ileus, intestinal, or colonic pseudo-obstruction, and idiopathic megacolon. In functional obstruction, patients present with lumen distention and obstructive symptoms similar to mechanical obstruction. However, there is a lack of apparent physical occlusion or obstruction distal to the involved bowel segment (De Giorgio et al., 2011; Wells et al., 2017).

The main symptoms of OBD are abdominal pain, distention, nausea, vomiting, and constipation (Gore et al., 2015). Gut motility dysfunction may be responsible for most of these symptoms including nausea, vomiting, distention, and constipation (Summers, 1999; Sarna and Shi, 2006; Shi et al., 2011). Accordingly, motility has been the primary focus of investigations into the pathophysiology of OBD. Abdominal pain is the primary reason for hospital visits in obstruction. In fact, mechanical BO composes 15% of all emergency admissions for abdominal pain (Cappell and Batke, 2008; Gore et al., 2015). Abdominal pain may be the only prominent symptom in patients with intestinal pseudo-obstruction (Hanauer and Wald, 2007; De Giorgio et al., 2011). However, mechanisms of abdominal pain in OBD have not been fully identified.

In the acute phase of BO (i.e., the first 12–24 h), abdominal pain is usually colicky (cramping and intermittent). The pain may increase, peak, and subside periodically (Summers and Lu, 1991; Cartwright and Knudson, 2008; Gore et al., 2015). However, patients with chronic partial obstruction may experience persistent abdominal pain (Ripamonti et al., 2001; Ripamonti and Mercadante, 2004). Among patients with malignant obstruction, 92% have distention-associated chronic abdominal pain (Baines et al., 1985). Current analgesic management for BO-associated pain relies on high doses of opioids (Ripamonti and Mercadante, 2004; Roeland and von Gunten, 2009). However, opioids are notoriously known to cause further bowel dysfunction, such as constipation and narcotic bowel syndrome (Grunkemeier et al., 2007; Ketwaroo et al., 2013). It is imperative to better understand mechanisms of abdominal pain in BO, in order to identify therapeutic targets specifically for distension-associated pain. In this review, we attempt to critically review the mechanisms for acute colicky pain and chronic abdominal pain in obstruction by focusing mainly on mechanical BO. Recent preclinical studies demonstrate that visceral hypersensitivity is present in chronic obstruction (Huang and Hanani, 2005; Lin et al., 2017b). We will offer novel insights into the mechanisms of visceral hypersensitivity in obstruction.

## Neurobiological Basis of Visceral Pain

In BO, abdominal pain is largely of visceral origin except in severe complications, i.e., peritonitis, where the peritoneum is involved. Unlike somatic pain, visceral pain is often dull, poorly defined, and localized. Visceral pain is usually referred to the overlying skin of the abdominal wall. Referred pain is thought to occur from convergence of somatic and visceral afferents upon the sensory neurons in the same spinal segments (Malykhina, 2007; Sengupta, 2009). For example, pain in LBO may be referred to the mid or hypogastrium (Huang and Hanani, 2005; Lin et al., 2017b). The visceral sensory receptors (nociceptors) are widely located on serosal, muscularis, and mucosal layers (Gebhart, 2000; Brierley and Blackshaw, 2006; Sengupta, 2009). Upon activation of nociceptors by mechanical or chemical stimuli, primary afferent nerves, traveling mainly with the splanchnic and pelvic sensory afferents, transduce the signals to the neuronal cell body located in dorsal root ganglia (DRG). This constitutes the peripheral processing of visceral sensation. The DRG neurons then synapse with second order neurons in the spinal cord to initiate central processing of sensory information. Further ascending visceral nociceptive information travels by spinothalamic pathways and ipsilateral and dorsal spinal pathways (Cervero, 2000; Sengupta, 2009; Greenwood-Van Meerveld et al., 2015).

It is now well recognized that visceral sensitization of the peripheral and central pathways is a key mechanism for abdominal pain and discomfort (Bielefeldt, 2006; Grundy et al., 2006; Azpiroz et al., 2007; Zhou et al., 2012). Several peripheral mediators, including neurotrophins (NT) such as nerve growth factor (NGF) and brain-derived neurotrophic factor (BDNF), prostaglandins such as PGE_2_, and 5-hydroxytryptamine (5-HT), may sensitize the processes of sensation and transduction of the afferent neurons by lowering nociceptor firing thresholds (Bielefeldt, 2006; Pezet and McMahon, 2006). The process of peripheral sensitization is critical to the development of visceral pain in GI disorders (Bielefeldt, 2006; Grundy et al., 2006; Greenwood-Van Meerveld et al., 2015).

## Acute Abdominal Pain in Bowel Obstruction

**FIGURE 1:**
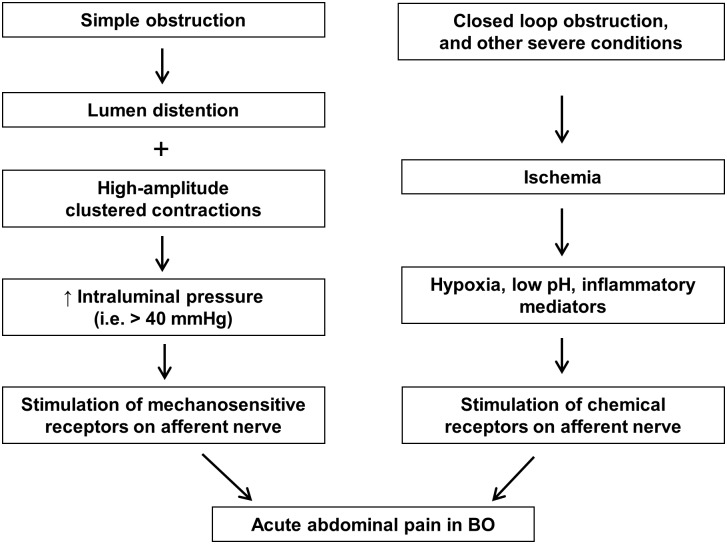
Nociceptive mechanisms involved in acute abdominal pain in bowel obstruction. In simple obstruction (left), colicky pain may be related to direct activation of mechanosensitive receptors in visceral afferents by increased intraluminal pressure associated with clustered contractions. In severe conditions such as closed loop obstruction (right), blood supply may be compromised in the affected area. Hypoxia, low pH, and inflammatory mediators released in the ischemic tissues may activate visceral afferents via chemical receptors, thus contributing to abdominal pain.

Abdominal pain may begin soon after obstruction occurs, and is usually associated with abdominal distention. The pain is more severe when obstruction occurs in the lower GI tract, especially in the colon. In the acute phase of BO, the pain is usually colicky (Summers and Lu, 1991; Gore et al., 2015). The exact nociceptive mechanisms of acute pain in BO are not well elucidated. However, it is thought that acute pain may be related to activation of mechanosensitive nociceptors in visceral afferents by increased intraluminal pressure associated with clustered contractions. In extreme cases such as closed loop obstruction, ischemia and released inflammatory mediators may further activate visceral afferents via chemical receptors, thus contributing to acute abdominal pain (**Figure 1**).

### Motility Changes and Colicky Pain in BO

Gut motility is markedly changed in response to either SBO or LBO (Summers and Lu, 1991). In the SBO, motility in the segment distal to occlusion is inhibited, whereas motor activity in the segment proximal to occlusion is temporarily increased after obstruction (Summers et al., 1983; Prihoda et al., 1984). This may be a result of enhanced peristalsis, as intraluminal mechanical retention at the site of obstruction stimulates neural response to cause ascending excitation of contractile activities (in the proximal segment) and descending inhibition (in the distal segment). Typically, clustered contraction patterns occur every few minutes in the distended proximal segment, and these contractions often are of high amplitude and propagate very fast at nearly 1 cm/s (Summers et al., 1983). Motor activity is also increased in the early hours of LBO, especially if obstruction occurs in the distal colon (Summers, 1999). These contractile activities will substantially increase intraluminal pressure in the segment oral to the occlusion. Under physiological conditions, the normal intraluminal pressure in the gut is nearly 0 cm H_2_O (Summers, 1999; Silen, 2005). In simple obstruction, the intraluminal pressure increases to 8∼10 cm H_2_O in the segment proximal to the site of obstruction. However, when high amplitude and rapidly propagating contractions occur, the intraluminal pressure may increase to over 40 mmHg transiently in the obstructed bowel (Shikata et al., 1983; Summers et al., 1983; Silen, 2005). The pressure increase, especially the one associated with high amplitude clustered contractions, may directly stimulate mechano-receptors of the sensory neurons, possibly leading to pain (**Figure 1**). In a human manometry study, Summers et al. (1983) recorded motility changes in a 30 cm long small bowel segment in patients with partial BO. They noticed that some of the clustered contractions in the recorded segment were directly associated with colicky pain, but others were not. They did not study the pressure–pain response relationship in the patients. However, it is possible that pressure increases over the threshold for noxious stimulus (i.e., ≥40 mmHg) may be required to initiate nociceptor activation (Ness et al., 1990; Lin et al., 2015). It is also known that the pressure–response relationship may be very different in different regions of the GI tract and among different classes of afferent fibers (Brierley and Blackshaw, 2006). Several mechanoreceptors in the visceral afferents have been identified in the GI tract: the degenerin/epithelial sodium channel (DEG/ENaC) family, the transient receptor potential (TRP) family of ion channels, i.e., TRPV4, and the recently identified Piezo channels (Brierley and Blackshaw, 2006; Volkers et al., 2015). Further investigations into the mechanotransduction signaling in the visceral afferents may help to delineate the mechanisms of colicky pain in BO.

It is also of notice that clustered high amplitude contractions are usually intercepted by a few minutes of quiescent period. During the quiescent period, patients usually feel pain free (Summers, 1999). This further indicates that the augmented motor activity may be the main reason for the colicky pain in obstruction.

### Ischemia and Abdominal Pain in BO

An increase in intraluminal pressure had been thought to cause local ischemia in the obstructed bowel. If so, ischemia might also play a role in acute abdominal pain in BO. However, it was later found that intraluminal pressures only reach 8–10 cm H_2_O in simple obstruction and are not high enough to cause transmural ischemia in the gut (Shikata et al., 1983; Summers and Lu, 1991). Even in the case of ischemia colitis, ischemic change is typically caused by a global reduction in blood flow to the colon, not by local obstructive lesions (Oglat and Quigley, 2017). Nevertheless, blood supply can be compromised through external compression of the bowel or its mesentery by severe adhesions, torsion, or intussusception. Then, abdominal pain becomes more prominent. Strangulation-associated ischemia of the gut wall may also be found in closed loop obstruction (Tavakkoli et al., 2014; Gore et al., 2015). If left untreated, severe complications such as perforation and peritonitis may occur. In these extreme cases, hypoxia, low pH, and inflammatory mediators released in the ischemic tissues or in peritonitis may stimulate chemical receptors and activate afferent neurons (**Figure 1**). Acid-sensitive ion channels (ASIC), TRPV1, and P2X3 are among many of the chemical receptors in the sensory nerve endings (Bielefeldt, 2006; Brierley and Blackshaw, 2006). Reviews on inflammation and ischemia associated abdominal pain can be found elsewhere (Vermeulen et al., 2014; Feuerstadt and Brandt, 2015; Oglat and Quigley, 2017).

If obstruction continues for longer than 12 or 24 h either in SBO or LBO, gut motor activity becomes gradually suppressed (Summers and Lu, 1991; Shi et al., 2011; Gore et al., 2015). Interestingly, as the initial increase in motility is replaced by reduced activity, so is the acute colicky pain replaced by a more chronic persistent pain (Fraser, 1984; Tavakkoli et al., 2014). This pain is a major concern in patients with chronic partial obstruction, such as inoperable or malignant obstruction (MBO) (Ripamonti et al., 2001; Ripamonti and Mercadante, 2004). Pre-clinical studies have revealed that visceral sensitivity is heightened within 72 h of obstruction (Huang and Hanani, 2005; Lin et al., 2017b). Visceral hypersensitivity may account for chronic abdominal pain in OBD. In the following parts of the review, we will focus mainly on the mechanisms of visceral hypersensitivity in BO.

## Mechanical Stretch-Induced Gene Expression of Pain Mediators in Bowel Obstruction

Lumen distention is a cardinal characteristic of OBD (Russell and Welch, 1990; Summers, 1999; Postier and Squires, 2007). It represents a circumferential stretch to the gut wall (Shi, 2017). Mechanical stretch may play a key role in abdominal pain in chronic obstruction such as MBO, because medications to release distention also reduces abdominal pain (Pandha and Waxman, 1996; Bicanovsky et al., 2006). In the past few years, we tested a hypothesis that mechanical stretch in lumen distention induces gene expression of pain mediators in the gut wall, which act on nociceptors to sensitize afferent neurons and contribute to visceral hypersensitivity and abdominal pain in OBD.

In an attempt to understand the scope of impacts of mechanical stretch on gene expression *in vivo*, we used a rat model of partial colon obstruction to screen for “stretch-sensitive” genes in a comprehensive Affymetrix cDNA array analysis (Shi et al., 2011; Shi, 2017). Overall, we identified several major groups of genes whose expression is altered by mechanical stretch, including those encoding certain inflammatory mediators, growth factors, neurotrophins, adhesion molecules, extracellular matrix proteins, and some cell signaling proteins (Shi et al., 2011). Of particular importance, we found that gene expression of several well-recognized pain mediators, i.e., cyclo-oxygenase-2 (COX-2), NGF, BDNF, was significantly up-regulated in the distended colon.

Focusing on mechano-transcription of COX-2 in BO, we found that mechanical stretch induces gene expression selectively in gut SMC (Shi et al., 2011; Shi, 2017). The levels of COX-2 mRNA and protein in the muscularis externa were dramatically increased in the stretched colon segment oral to obstruction, but not in the non-stretch aboral segment. Immunohistochemical studies in the full-thickness colon specimens showed that the increased COX-2 expression occurs selectively in the circular and longitudinal SMC, but not in the mucosa, submucosa, or myenteric plexus (Shi et al., 2011). Thus, mechano-transcription of COX-2 in BO is a smooth muscle specific phenomenon. Choudhury et al. (2015) found that SMC specific α-actin with an intact intracellular network is required for mechano-transcription of COX-2 in colonic SMC. Li et al. (2012a) further studied the intracellular mechanisms of mechano-transcription of COX-2 in gut SMC, and found that mitogen-activated protein kinase (MAPK) ERK, p38, and JNK, and protein kinase C (PKC) especially PKCdelta, are important parts of signaling cascade in mechano-transcription of COX-2 (Li et al., 2012a,b; Shi, 2017).

**FIGURE 2:**
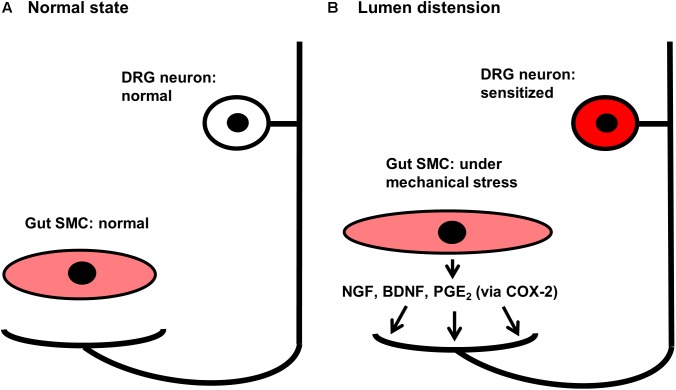
Proposed mechanisms of visceral hypersensitivity in bowel obstruction. **(A)** Gut SMC and DRG neuron in a normal state. **(B)** Gut SMC and DRG neuron in bowel obstruction. Note that lumen distention-associated mechanical stretch induces gene expression of pain mediators NGF, BDNF, and COX-2 in gut SMC in obstruction. The mechanical stretch-induced pain mediators act on nociceptors in the gut, and sensitize DRG neurons by altering gene expression and channel functions. This may contribute to abdominal pain in chronic bowel obstruction. SMC, smooth muscle cells; DRG, dorsal root ganglia; NGF, nerve growth factor; BDNF, brain-derived neurotrophic factor; PGE_2_, prostaglandin E_2_; COX-2, cyclo-oxygenase-2.

Lin et al tested if colon distention-induced COX-2 plays a role in visceral hypersensitivity (Lin et al., 2015). Sub-noxious tonic distention of the distal colon with a balloon at 20∼40 mmHg for 40 min induced COX-2 expression and PGE_2_ production in colonic smooth muscle, but not in the mucosa layer. Lumen distention also increased visceral sensitivity. The visceral hypersensitivity persisted for at least 3 days. Electrophysiological studies found that the excitability of colon projecting sensory neurons in the DRG was markedly augmented. Nevertheless, administration of COX-2 inhibitor NS-398 blocked distention-induced production of PGE_2_ (Lin et al., 2012), and significantly attenuated visceral hypersensitivity (Lin et al., 2015). These data thus suggest that lumen distention-induced expression of pain mediators, i.e., PGE_2_, may contribute to visceral hypersensitivity in conditions with lumen distention (**Figure 2**).

## Pathophysiological Role of Mechanical Stress-Induced Neurotrophins in Visceral Hypersensitivity in Obstruction

Neurotrophins such as NGF and BDNF are well-recognized pain mediators (Bielefeldt, 2006; Pezet and McMahon, 2006). In the rat model of BO, Lin et al. (2017b) found that the expression of NGF mRNA and protein was increased in colonic SMC in the distended colon oral to obstruction, but not in the segment distal to the site of obstruction. Mechanical stretch *in vitro* also led to robust expression of NGF mRNA and protein in colonic SMC. In obstruction rats, the cell excitability of colon-projecting DRG neurons was significantly enhanced. Moreover, the referred visceral sensitivity was enhanced in BO, as the withdrawal response to von Frey filament stimulation to the lower abdominal wall was markedly increased in BO rats. The *ex vivo* study showed that DRG neuron excitability was enhanced significantly by incubation with media collected from the muscle strips of the oral distended segment of BO rats, but not by media from sham controls or the aboral non-distended segment of BO rats. Moreover, the increased cell excitability was significantly attenuated by anti-NGF antibody, suggesting that mechanical stress-induced smooth muscle-derived NGF plays a critical role in colon neuron sensitization in BO (**Figure 2**). Further *in vivo* studies found that treatment with anti-NGF antibody attenuated colon neuron hyper-excitability and referred hypersensitivity in BO rats. Obstruction also led to a significant increase of tetrodotoxin-resistant (TTX-r) Na^+^ currents and up-regulation of mRNA expression of TTX-r Na_v_1.8, but not TTX-sensitive Na_v_1.6 and Na_v_1.7 in colon-projecting sensory neurons. These changes were abolished by anti-NGF treatment (Lin et al., 2017b). Thus, stretch-induced NGF in colon SMC plays a critical role in visceral hypersensitivity in BO, by acting on TTX-r Na^+^ channels in sensory nerve (**Figure 2**).

Expression of BDNF mRNA and protein was also increased dramatically in the colonic smooth muscle of the oral dilated segment, but not in the non-dilated segment aboral to obstruction (Lin et al., 2017a; Fu et al., 2018). There was an eightfold increase in BDNF mRNA expression in colonic smooth muscle on day 3 in BO. Compared with sham controls, colon neurons of the BO rats demonstrated significantly reduced densities of total K^+^ current and the transient inactivating A-type (*I_A_*) current, but not the delayed rectifier current (*I_K_*). The mRNA expression of *I*_A_ subtype K_v_1.4 in colon neurons was down-regulated in BO. Treatment with anti-BDNF antibody *ex vivo* restored total K_v_ and *I*_A_ currents of neurons from BO rats. Administration of inhibitor of BDNF receptor (Trk B) ANA-12 blocked BO-associated changes of neuronal excitability, K_v_ activity, and gene expression in obstruction. This is associated with improvement of referred hyperalgesia (Lin et al., 2017a). Thus, mechano-transcription of BDNF in gut SMC contributes to visceral hypersensitivity in BO mainly by suppressing A-type K^+^ currents and gene expression in the primary sensory neurons (**Figure 2**).

## Other Possible Mechanisms and Future Perspectives

Huang and Hanani (2005) found that the coupling among satellite glia cells in DRG was enhanced by nearly 18-fold in chronic intestinal obstruction in mice. Histological studies found that the size of DRG neurons innervating the obstructed segment in the gut was increased in prolonged obstruction (3 weeks or longer) (Williams et al., 1993; Huang and Hanani, 2005). The mechanisms and significance of the DRG morphological changes in obstruction are not known. In our model of partial colon obstruction, we did not find significant changes in the size of DRG neurons in obstruction up to 7 days (Lin et al., 2017b). However, Huang and Hanani proposed that these morphological changes may contribute to abdominal pain in BO (Huang and Hanani, 2005), as increased glial coupling has the potential to mediate long-distance communications between sensory neurons (Slugg et al., 2000).

It is yet to determine whether other peripheral mechanisms and central sensitization are involved in the pathogenesis of abdominal pain in OBD. Mechanical stretch in obstruction may cause injury and disruption in enteric neurons and interstitial cells of Cajal (Chang et al., 2001; Wedel et al., 2002; Wu et al., 2013; Lin et al., 2014). The mechanisms underlying these effects deserve further investigation, as injuries of enteric nerves may contribute to sensory-motor dysfunction in OBD. Whether mechanical stretch affects extrinsic afferent nerve endings in obstruction remains to be determined. If so, this may contribute to visceral sensitivity in OBD. Investigation into the signaling mechanisms of mechano-transcription of pain mediators in the gut has commenced (Li et al., 2012a). Further studies may help to identify therapeutic targets to block the mechano-transcription process for the management of abdominal pain in OBD.

## Conclusion

In summary, multiple mechanisms may account for abdominal pain in BO. While augmented motility changes are associated with acute colicky pain in simple obstruction, ischemia and an increased release of inflammatory mediators may contribute to acute pain in severe conditions such as closed loop obstruction. Visceral sensitivity is increased in BO, and visceral hypersensitivity may underlie the mechanisms of chronic abdominal pain in BO. Mounting evidence suggests that mechanical stretch-induced expression of the nociceptive mediators NGF, BDNF, and COX-2 in the distended bowel plays a critical role in the development of visceral hypersensitivity in BO.

## Author Contributions

X-ZS initiated the first draft of the manuscript. X-ZS, Y-ML, and SH all contributed to the final version of the review, and approved it for submission and publication.

## Conflict of Interest Statement

The authors declare that the research was conducted in the absence of any commercial or financial relationships that could be construed as a potential conflict of interest.
